# Research and Remedies

**Published:** 1913-04-19

**Authors:** 


### RESEARCH AND REMEDIES.

### Delayed Chloroform Poisoning.

Although this title is undoubtedly a misnomer, since the condition follows ether and ethyl chloride as well as chloroform, any light upon its pathology is of value. In the *Johns Hopkins Hospital Bulletin* Mosiman and Whipple detail the result of their investigations upon certain animals which have nucleated red corpuscles. Thus they find that the pigeon, frog, and terrapin are practically immune from those characteristic hepatic degenerations which are so easy to produce in mammals by prolonged or repeated chloroformisation. Human fœtuses and young puppies display a similar but less marked power of resistance, and in these there are large quantities of nucleated cells in intimate association with the liver-cell columns; these nucleated cells never show any evidence of injury after chloroform administration. Chloroform, unlike phosphorus, is essentially a nuclear poison. There is a great temptation, say the authors, to correlate the protection of the liver cells against chloroform with the presence of these cells, though they admit that the exact mechanism is not clear.

### The Heart and Pulse in Pregnancy.

The respective effects of heart disease upon pregnancy, and of pregnancy upon heart disease, are always very difficult to forecast in any individual case; and the most divergent views exist, probably because few obstetricians see personally more than a few cases of the combination. Tuszkai, of Marienbad, publishes a long paper on the subject in the *American Journal of Obstetrics and Gynæcology*; and without laying down definite rules of prognosis, makes some interesting observations. In normal women, he says, there is a variation in the pulse rate, between the sitting and supine positions, of several beats per minute. In normal pregnancy this variability disappears; and Graves, he says, regarded this as a most characteristic confirmatory sign of pregnancy. In pregnancy complicated by heart disease, the variability does not disappear, or, if it does, it soon returns in increased degree, combined with symptoms due to insufficient compensation. He regards the prognosis of this class of case as grave; although he admits, what every practising obstetrician knows, that many women with marked heart lesions get through pregnancy and labour without any obvious ill-effects. As to whether gestational heart disease (*cardiopathie de la grossesse*) actually exists or not he will not definitely commit himself; but the impression got from reading his paper is that he is inclined to admit it, though unable to advance a strict proof.

### A New Sign of Sinus Thrombosis.

The signs by which thrombosis of the sigmoid sinus of the brain is diagnosed are so inconclusive that too often this serious emergency escapes recognition until its consequences are past repair; and, on the other hand, they may suggest this condition when in reality some other lesion—pneumonia, malaria, meningitis, etc.—is the cause of the symptoms. Any aid, therefore, in diagnosing the onset of sinus thrombosis at the earliest moment will be of great service to surgeons and patients. Crowe describes such a sign in the *Johns Hopkins Hospital Bulletin*, based upon a most ingenious rationale. This observer satisfied himself that if, in a healthy person, one internal jugular vein is compressed in the neck, the retinal vessels show no change; but when both sides are thus obstructed, the retinal veins dilate owing to stasis following an obstructed outflow. If then one jugular vein (or the sigmoid sinus from which it arises) is blocked by clot, pressure on the vein of the opposite side will produce retinal venous dilation; whereas if both jugulars are patent, pressure on one of them will produce no such result. The test is especially valuable if in the early course of any disease in which sinus thrombosis is a possible complication the jugulars are found to be both patent, but subsequently one of them becomes obstructed. The author has put his idea to the proof clinically in a series of cases, and has found it to answer admirably. In several cases thrombosis was thus discovered and appropriately treated when otherwise it would not have been suspected, or at least would have been very doubtful.

